# Effectiveness of Telerehabilitation for Correcting Posture in Elderly with Thoracic Kyphosis in Urban Thailand

**DOI:** 10.5195/ijt.2023.6566

**Published:** 2023-12-12

**Authors:** Santhanee Khruakhorn, Nathaphon Jirasakulsuk, Pattaridaporn Saengpromma

**Affiliations:** Department of Physical Therapy, Faculty of Allied Health Sciences, Thammasat University, Thailand

**Keywords:** Cost-effectiveness, Elderly, Exercise, Telerehabilitation, Thoracic kyphosis

## Abstract

**Introduction:**

Thoracic kyphosis (TK) is an abnormal thoracic spine. Telerehabilitation and conventional physical therapy were compared for correct posture in the thoracic angle, forward head posture, back muscle strength, and cost-effectiveness.

**Method:**

Twenty-two Thai women 60 years of age and over, with thoracic angles over 45 degrees, participated in this study. Participants were randomly allocated into a telerehabilitation group (TG) or a control group (CG). TG and CG underwent a thoracic corrective exercise program for 1 hour each session, three times per week for 8 weeks.

**Result:**

Thoracic angle, forward head posture, and back muscle strength improved (P < .05) in both groups. Furthermore, cost-effectiveness showed the cost of intervention in TG lower than CG, approximately 133.78 US$.

**Conclusion:**

Our study showed no difference in telepractice program efficacy and effectiveness compared to in-person treatment in the clinic. Thus, telerehabilitation may be an alternative treatment for the elderly who cannot travel to the hospital.

Thoracic kyphosis (TK) is an abnormality of thoracic spine alignment. TK causes a thoracic angle higher than 40 degrees, leading to spine misalignment forward bending, spine inclination, or flex posture (Imagama et al., 2014; Takahashi et al., 2005). This problem could result in abnormal postures such as the forward head (Singla & Veqar, 2017) and the rounded shoulder (Singla & Veqar, 2017). Age-related TK was most common when people reached the age of 60 or retirement; an estimated 20–40% of the elderly had TK (Imagama et al., 2014; Kado et al., 2014; Tokida et al., 2019). TK affects the daily living difficulties of the elderly due to back muscle weakness, decreased walking distance, difficulty climbing stairs (Katzman et al., 2015; Tokida et al., 2019), and an increased risk of falling (McDaniels-Davidson et al., 2018).

Many TK interventions in the elderly (Bautmans et al., 2010; Greendale et al., 2009; Katzman et al., 2007), such as manual therapy (Bautmans et al., 2010), taping technique (Bulut et al., 2019), thoracic bracing (Pfeifer et al., 2004), and modalities (Filiz & Firat) has been proved to be effective—especially the correct posture exercise (Bansal et al., 2014). Correct posture programs focus on increased joint mobility, muscle strength, endurance, and maximized activity capacity. The program assists with learning the correct posture by keeping the muscles functioning correctly. The proprioceptive receptors are stimulated to adjust their posture (Bansal et al., 2014; Jang et al., 2015; Kado et al., 2009; Katzman et al., 2007). The rehabilitation period varied in studies; over six months (Katzman et al., 2017), twelve weeks (Greendale et al., 2009; Katzman et al., 2007), and eight weeks (Jang et al., 2015, 2019; Katzman et al., 2017). This rehabilitation enhanced physical capacity, balance, and quality of life by changing the angle of the thoracic spine (Greendale et al., 2009; Jang et al., 2015, Jang et al., 2019; Katzman et al., 2007; Katzman et al., 2017).

Telerehabilitation is used across the world and employs various types of communication. Previous studies on telerehabilitation have explored text messages, phone calls, video conversations, and mobile applications. Some patients were not required to see a specialty doctor at the hospital but were still treated (Fukuoka et al., 2015). Telerehabilitation increased the continuity of healthcare visits (Sumriddetchkajorn et al., 2019), and reduced the possibility of missing an appointment due to traffic, higher cost, time for waiting, and sick leave from work (Wattanapisit & Saengow, 2018). Therapies that include telerehabilitation, have been growing increasingly widespread and are improving health systems' accessibility,

A previous study showed that a home program increased exercise constancy in the elderly (Bean et al., 2019). Participants could be asked questions and receive immediate advice. Real-time communication improves self-care and exercise confidence (Lawford et al., 2019).

Telerehabilitation is a relatively new service delivery model not yet studied in Thailand. This study aimed to compare the effectiveness of telerehabilitation and exercise under physical therapy supervision for correcting posture in the thoracic kyphosis angle, forward head posture, and back muscle strength in elderly with TK. A secondary objective was to determine the cost-effectiveness of telerehabilitation compared to exercise under physical therapy supervision. The overall goal was to increase the therapeutic potential and improve the quality of life of the elderly.

## Methods

### Study Design

The telerehabilitation study is a single-center, single-blind, randomized controlled trial conducted between December 2021 and May 2022. The study protocol was approved by The Human Research Ethics Committee of Thammasat University (Science) (reference number: 041/2564) and registered in Thai clinical trials: TCTR20210719004.

### Population and Randomization

Twenty-seven participants enrolled from the Physical Therapy and Hydrotherapy Unit, Faculty of Allied Health Sciences, Thammasat University. The inclusion criteria were thoracic kyphosis angle over 45 degrees, flexible kyphosis angle of at least 5 degrees, walking independently, and having a smartphone. The exclusion criteria were mild cognitive impairment, severe medical conditions limiting exercise, history of fracture, history of hip or knee replacement, regular exercise, and not passing the safety test. The sample size was calculated via G*Power 3.1.9.4, a program for calculating the sample size. Calculations were estimated using the F-test statistics with the following values: Effect size = 0.48, Power = 0.95, Level of significance = 0.05 (Jang et al., 2015). Participants were randomly assigned into one of two groups: a telerehabilitation group of 11 participants and an exercise-in-clinic group of 11 participants. The statistician formed groups by computerized simple random sampling.

### Outcomes Measurement

This study had two physical therapists. The first was the assessor, who responded to all measurements at baseline and, after 6 weeks of training, was blinded for the participant's group. The other physical therapist conducted the training sessions for both groups.

### Thoracic Kyphosis Angle

The thoracic kyphosis angle was assessed in degrees using the Dual Digital Inclinometer (Lafayette ACU002 Acumar Dual Inclinometer) while the participant stood comfortably. The high degrees indicated that TK was severe (Kado et al., 2014). The accuracy and reliability were excellent (0.86 and 0.89, respectively). The first researcher explained the suitable standing posture. The participants prepared for the best comfortable stand by following the instructions, “Swing your arms forward and back, bend and extend the neck, and take a full breath.” The thoracic kyphosis angle was assessed during one trial at baseline and after the training session.

### Forward Head Posture

The tragus-to-wall test indicated forward head posture (Heuft-Dorenbosch et al., 2004). Participants stood in a relaxing neck posture, eyes looking forward, hip and back placed against the wall, and feet placed 10 cm. away from the wall. The square rule was used for measuring the distance from the wall to the tragus of the ear. The distances in centimeters from both sides were measured once, and the average of both sides' distances was recorded.

### The Back Muscle Strength

The back muscle strength (Bergström et al., 2011) was assessed by a digital back-leg-chest dynameter (T.K.K.5402, Takei Co., Japan) (Imagama et al., 2011) which is an isometric contraction test in a standing position. The participants wore comfortable clothing and took off their shoes while standing, bent their hips at 30 degrees from the vertical line while their back kept straight, and both hands held the handle that placed the front of knees with straight arms and knees. The participants extended their back straight and held for 3 seconds, resting for 2 minutes. The average of three times was recorded in kilograms. The participants performed one practice session before taking the actual measurement.

### Cost Analysis

The cost of accessing the health system was recorded based on the cost of access: cost of medication, exercise, physical therapy, total duration, transportation costs, internet cost, mobile cost, and such expenses did not include treatment in other areas such as medical treatment, emergency symptoms, including receiving self-treatment such as purchasing self-medication. The costs were recorded for every physical therapy session. The results were analyzed by comparing the results of two treatments as follows.

A cost-effectiveness analysis was analyzed to estimate the cost of having a more remarkable thoracic kyphosis angle change. The calculated cost reflects the value needed to treat the condition and compares the two types of intervention by Incremental cost-effectiveness analysis. The cost of telerehabilitation and exercise in the clinic is compared by the post-study difference of the two intervention models to determine the cost of treatment compared to the thoracic kyphosis angle.



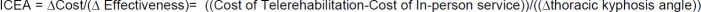



### Intervention Group

All participants explained the correct posture exercise protocol and practiced with the exercise tool in a trial session after baseline evaluation. The exercise protocol was used to improve muscle and spine mobility, increase strength and right postural awareness, and consisted of 1-hour sessions, 3 days per week, every other day for 8 weeks. Correct posture recognition was routinely recorded in a logbook. The correct posture exercise program would last 60 minutes, including 5 minutes of warm-up, 45 minutes of workouts (stretching, strengthening, self-mobilization, and postural control), and 5 minutes of cool-down.

In addition, participants learned methods and practices for using telecommunication equipment via video tutorials that explained the wireless headphone connection to a smartphone, how to adjust and set the camera on a tripod, and how to install the mobile application. The Line application was used for video conferencing through the exercise session, chatting about appointments, or answering questions.

Participant safety was a priority. A physical therapist recorded the address and contact number of each caregiver and taught them first aid methods for potential adverse events such as pain from exercise, falls, and the assessment of fractures. The researchers were to promptly contact caregivers and emergency services if an incident occurred.

### Control Group

Participants in the Control group received in-person services with a physical therapist. The physical therapist advised correcting posture without touching feedback and including encouraging exercise during exercise programs.

### Statistical Analysis

The variables were collected as mean and standard deviation using SPSS, version 22.0 (IBM® SPSS® Statistics is a powerful statistical software platform). The Shapiro-Wilk test was used for analyzing the pre-evaluation data to determine the distribution of the data. The Two-Way Mixed ANOVA test analyzed the differences within-group and between-groups. The analysis would also be assessed by setting the statistical significance level p <0.05. Additionally, the modified intention-to-treat (mITT) was used to assume altered results from pre-trial when data was lost during follow-up or pre-study withdrawal. If more than five exercise deficiencies occurred, the data were not analyzed in this study.

## Results

**Figure 1.**
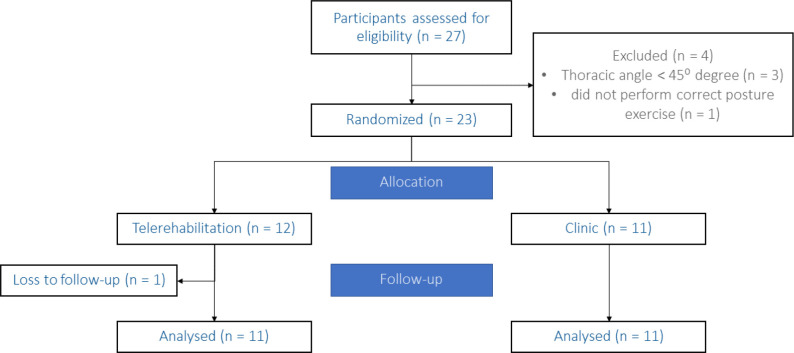
Flow Diagram

The recruitment of 27 participants occurred between December 2021 and May 2022. Three individuals satisfied the eligibility criteria for kyphosis (Kyphosis 45 degrees), and one did not execute correct posture exercises due to underlying conditions. However, twenty-three participants met all eligibility criteria and enrolled in the study. The intervention group received twelve individuals, whereas the control group had eleven. One participant in the telerehabilitation group did not complete the study during the correct posture exercise because she was occupied and could not continue the study. The eleven participants in both groups completed the intervention and follow-up visit. This sample included 22 women (Figure 1). The intervention group's mean age, height, and weight (n=11) were 66.55 ± 4.20 years, 153.55 ± 5.42 cm., and 55.09 ± 8.83 kg, respectively. Whereas the values in control group were 65.73 ± 3.29 years, 157.00 ± 5.29 cm., and 61.36 ± 7.98kg.

**Table 1.** Demographic Characteristics (n = 22)

Characteristic	Telerehabilitation group (n = 11)	Clinic group (n = 11)
Gender (male/female), N (%)	0 (0%)/11 (100%)	0 (0%)/11 (100%)
Age, yrs	66.55 ± 4.20	65.73 ± 3.29
Height, cm	153.55 ± 5.24	157.00 ± 5.29
Weight, kg	55.09 ± 8.83	61.36 ± 7.98
BMI (Body mass index)	23.34 ± 3.42	24.99 ± 3.89

### Effect of Telerehabilitation

There was no significant difference between the telerehabilitation and conventional group at baseline and post-assessment in all variables. Moreover, there was no significant difference in the group x time interaction at baseline and post-assessment for both groups. Thus, there was no differential effect between the intervention and control group between baseline and post-assessment.

### Effect of the Correct Posture Program

All clinical variables improved significantly after exercise sessions in both groups. Thoracic kyphosis angle improved in both groups (p < 0.01; the mean difference between the intervention and control group was 6.46 and 6.09, respectively). The same improvements in the intervention and control group were observed for forward head posture (p < 0.05; 1.14 cm. and 1.22 cm., respectively) and back strength (p < 0.01; 8.19 kg. and 7.38 kg., respectively). See Table 2.

**Table 2.** Mean of Outcomes Measure at Baseline and Post-test

Outcomes	Groups	Mean Baseline (95% Cis)	Mean at Post-test (95% Cis)	Time		Group	
				F (1, 20)	p	F (1, 20)	p
Kyphosis ankle	Telerehabilitation	52.73 (48.36, 57.10)	46.27** (42.17, 50.38)	62.36	0.01	0.36	0.95
	Clinic	52.73 (48.36, 57.10)	46.64** (42.53, 50.74)				
Forward posture	Telerehabilitation	13.21 (12.09, 14.32)	12.07* (11.21, 12.92)	16.91	0.01	0.01	0.97
	Clinic	13.27 (12.16, 14.39)	12.05** (11.19, 12.90)				
Back strength	Telerehabilitation	42.83 (34.41, 51.26)	51.02** (43.14, 58.89)	32.83	0.01	1.14	0.30
	Clinic	48.96 (40.53, 57.38)	56.34** (48.47, 64.22)				

* Significant difference between Baseline and Post-test (P < 0.05)

** Significant difference between Baseline and Post-test (P < 0.01)

### Cost Effectiveness

Each participant had 24 visits to the correct posture program. Average compliance was 89 ± 9% for the intervention group and 87 ± 5% for the control group. The cost analysis included first direct costs (physician cost, clinical equipment, income loss); second, indirect costs (cost of travel, fuel, and parking); and third, technology costs (internet cost, technology equipment). See Table 3.

**Table 3.** Cost Report

Cost category	Item	Telerehabilitation US$	%	Convention US$	%
Direct cost	Physician cost	370.50 ± 11.70	88.18	343.34 ± 7.54	61.85
	Clinical equipment	8.13	1.94	8.13	1.46
	The income loss	0	0	90.46 ± 59.03	16.29
Indirect cost	Travel to clinic	N/A		112.72 ± 24.33	20.31
Technological	Technical equipment	20.13	4.79	N/A	
	Internet costs	20.88 ± 2.80	5.09	N/A	
Total cost		420.15 ± 11.81	100	555.15 ± 67.81	100

The intervention group used 420.15 US$, while the control group used 555.15 US$. The direct cost was from physician cost, equipment, and income loss in the intervention group (370.50 US$, 8.13 US$, and 0 US$, respectively) and the control group (343.34 US$, 8.13 US$, and 90.46 US$, respectively). The intervention group did not pay for the indirect cost, such as traveling to health services, whereas the control group used an average of 112.72 US$. Technology cost was used for equipment and internet costs only in the intervention group (20.13 US$, and 20.88 US$, respectively). The cost-effectiveness analysis was analyzed to estimate the cost of thoracic kyphosis angle changes (see Table 4). Telerehabilitation reduced the incremental cost-effectiveness ratio more than the conventional group (−371.61 US$). Therefore, the cost-effectiveness was worthwhile for the telerehabilitation group's kyphosis angle change over 8 weeks.

**Table 4.** Incremental Cost-Effectiveness Analysis

Groups	Total Cost US$ (95% CI)	Incremental cost US$ (95% CI)	Effects, mean (95% CI)	Incremental effects, mean kyphosis angle (95% CI)	Incremental cost-effectiveness ratio of US$/Kyphosis angle
Telerehabilitation	420.15 (390.27, 442.44)	−133.78 (−276.06, 8.49)	6.45 (4.50, 8.27)	0.36 (−2.950, 3.678)	−371.61
Clinic	555.15 (400.42, 699.86)	N/A	6.09 (3.86, 8.25)	N/A	N/A

## Discussion

This study compared the effects of a posture correction program for thoracic kyphosis in the elderly between intervention and control groups. The results showed that the posture correction exercise improved the spinal alignment of older adults with TK. Moreover, the effectiveness of correcting posture program was similar between groups. The positive effect was on participant acceptance and satisfaction with the intervention. Based on participants' feedback, the exercise program intensity was enough, and participants were appreciated appreciative.

Likewise, a previous study showed the positive benefit of telerehabilitation in the elderly. Telerehabilitation via video demonstration with real-time text message feedback improved kyphosis angle, physical function, and quality of life. The kyphosis angle in the previous study had an 8-degree decrease (Katzman et al., 2019). Another prior study found that the joint range of motion had no differential effect between the telerehabilitation and clinic groups (Tousignant et al., 2011). In patients with chronic low back pain, telerehabilitation was used via the application for exercise 24 sessions (8 weeks) (Mbada et al., 2019). The result revealed that the telerehabilitation group was equal to clinic service in pain, activity limitation, general health status, and back muscle endurance. In addition, the present telerehabilitation delivered service used low-cost treatment compared to the effectiveness.

The cost-effectiveness of this study found the telerehabilitation group to be less expensive than the clinical group (133.78 US$). Similar to previous studies, telerehabilitation in lower back pain was less expensive than clinical service by a mean difference of about 44.26 US$ (Fatoye et al., 2020). The previous study on total knee arthroplasty showed better yearly cost savings in telerehabilitation than in clinic service (342.86 US$) (Fusco & Turchetti, 2016). The cost of travel for treatment was indirectly proportional to the number of treatment sessions, which is more expensive when compared to the cost of the internet in the telerehabilitation group. The telerehabilitation group spent time in intervention more than the clinic group, due to technology problems such as internet instability and the video view not being appropriate. In comparing the difference between the cost divided by the quality of intervention, it was found that the incremental cost-effectiveness ratio US$/Kyphosis angle was 371.61 US$.

According to previous studies (Chen et al., 2016), the success factors for telerehabilitation were divided into four main categories. First, the providers must be optimistic about telerehabilitation services, instill confidence in the treatment, and make proper treatment decisions. Providers should possess creative skills to resolve challenges and instill confidence. Second, the receiver's learning and experience level were necessary to accept the telerehabilitation service. Third, the provider's environment required sufficient space to demonstrate the movement of the rehabilitation program. The environment needed to be in a private zone without noise to enable them to provide clear advice to participants. Fourth, the participant's environment required internet and technical assistants, without which participants could not cope with technical problems.

This study was the first to evaluate the efficacy of telerehabilitation services on the elderly in Thailand. Our study showed that the effectiveness of telerehabilitation was equal to conventional service and less expensive (Jirasakulsuk et al., 2022). The results confirmed that telerehabilitation could be an additional therapeutic option for the elderly who had obstacles traveling to the hospital. Additional considerations should include efficacy and safety and the ability to learn and comprehend telerehabilitation. The participants should always be in contact with the therapy provider during the exercise session, and an onsite practice session must first be performed to ensure that the participants can understand and correctly implement all equipment.

A limitation of this study was that the screen size of participants' mobile phones was too small to clearly visualize the demonstrated correct exercise posture. Therefore, further studies should determine the appropriate screen size for the elderly. The generalizability to the population was limited because only female participants were recruited.

## Conclusion

Our study was Thailand's first on telerehabilitation in the elderly with thoracic kyphosis. The results discovered that when comparing the effectiveness of treatment between traditional services, there was no difference in the intervention's effect. As a result, telerehabilitation could serve as a model for future alternative treatment if elderly patients who have obstacles to getting service at the hospital. Telerehabilitation could also prevent missing therapeutic appointments because of unavailable caregivers.
